# Risk Classification for Overall Survival by the Neutrophil–Lymphocyte Ratio and the Number of Metastatic Sites in Patients Treated with Pembrolizumab—A Multicenter Collaborative Study in Japan

**DOI:** 10.3390/cancers13143554

**Published:** 2021-07-15

**Authors:** Taizo Uchimoto, Kazumasa Komura, Wataru Fukuokaya, Takahiro Kimura, Kazuhiro Takahashi, Yusuke Yano, Kazuki Nishimura, Keita Nakamori, Yuya Fujiwara, Tomohisa Matsunaga, Takeshi Tsutsumi, Takuya Tsujino, Ryoichi Maenosono, Yuki Yoshikawa, Kohei Taniguchi, Tomohito Tanaka, Hirofumi Uehara, Hajime Hirano, Hayahito Nomi, Kiyoshi Takahara, Teruo Inamoto, Shin Egawa, Haruhito Azuma

**Affiliations:** 1 Department of Urology, Osaka Medical and Pharmaceutical University, Osaka 569-8686, Japan; taizo.uchimoto@ompu.ac.jp (T.U.); uro089@osaka-med.ac.jp (Y.Y.); kazuki.nishimura@ompu.ac.jp (K.N.); keita.nakamori@ompu.ac.jp (K.N.); uro072@osaka-med.ac.jp (Y.F.); uro065@osaka-med.ac.jp (T.M.); uro070@osaka-med.ac.jp (T.T.); uro061@osaka-med.ac.jp (T.T.); uro064@osaka-med.ac.jp (R.M.); uro066@osaka-med.ac.jp (Y.Y.); hirofumi.uehara@ompu.ac.jp (H.U.); uro052@osaka-med.ac.jp (H.H.); uro022@osaka-med.ac.jp (H.N.); tinamoto@osaka-med.ac.jp (T.I.); uro001@osaka-med.ac.jp (H.A.); 2 Translational Research Program, Osaka Medical and Pharmaceutical University, Osaka 569-8686, Japan; sur144@osaka-med.ac.jp (K.T.); gyn123@osaka-med.ac.jp (T.T.); 3 Department of Urology, The Jikei University School of Medicine, Tokyo 105-8461, Japan; wfukuokaya@gmail.com (W.F.); h19ms-takahashi@jikei.ac.jp (K.T.); s-egpro@jikei.ac.jp (S.E.); 4 Department of Urology, Fujita-Health University School of Medicine, Nagoya 470-1192, Japan; takahara@fujita-hu.ac.jp

**Keywords:** pembrolizumab, platinum-refractory, overall survival, risk model, risk factor, neutrophil-lymphocyte ratio, number of metastatic sites

## Abstract

**Simple Summary:**

Pembrolizumab improves overall survival (OS) in patients with platinum-refractory metastatic urothelial carcinoma (mUC), whereas objective response was observed in a modest number of patients (<25%) for this treatment, implying the distinct survival outcomes for those patients. Thus, the optimal risk stratification to predict survival outcomes at the initiation of pembrolizumab treatment would be helpful for physicians. In the present study, we examined a risk model developed using two clinical factors, including the number of metastatic sites and neutrophil–lymphocyte ratio (NLR), for predicting OS at the initiation of pembrolizumab treatment. This risk stratification seemed to be well-balanced (26.5%, 44.3%, and 29.2% in the favorable-risk, intermediate-risk, and poor-risk groups, respectively), and Kaplan–Meier curves illustrated clear discrimination of OS among the risk groups. Since the model proposed in the present study can be concisely determined at the initiation of pembrolizumab treatment, physicians may be encouraged to consider the risk group for daily practice.

**Abstract:**

Pembrolizumab has emerged as the new standard of care in patients with platinum-refractory metastatic urothelial carcinoma (mUC), whereas the optimal risk stratification to predict survival outcomes is still controversial. We examined a risk model for overall survival (OS) in mUC treated with pembrolizumab using our multi-institutional dataset (212 patients). The median age was 72 years old. Median OS from the initiation of pembrolizumab treatment was 11.7 months. The objective response rate (ORR) was 26.4%. On multivariate analysis, multiple metastatic sites and an NLR > 3.50 at the initiation of pembrolizumab treatment were identified as independent predictors for OS. We next developed a risk model using those two predictors. Patients without any factors were assigned to the favorable-risk group (26.5%). Patients with either factor and both factors were assigned to the intermediate-risk group (44.3%), and poor-risk group (29.2%), respectively. Kaplan–Meier curves showed clear discrimination of OS among the risk groups (*p* < 0.001). The ORR in each group was 35.7% in the favorable-risk group, 27.7% in the intermediate-risk group, and 17.7% in the poor-risk group. Given that the model can be concisely determined at the initiation of pembrolizumab treatment, physicians may be encouraged to consider the risk group for daily practice.

## 1. Introduction

Platinum-based chemotherapy has been used as the first-line therapy for patients with metastatic urothelial carcinoma (mUC). Historically, since the GC (gemcitabine and cisplatin) regimen was approved by the Food and Drug Administration (FDA) with a similar effect for clinical survival and a lower rate of intolerable treatment-related adverse events (AE) compared with the conventional MVAC (methotrexate, vinblastine, doxorubicin, and cisplatin) regimen [1], GC chemotherapy has been considered as the standard of care in mUC patients. However, due to the lack of reliable second-line treatments after the treatment failure of the first-line therapy, a modest improvement of clinical survival was offered for mUC patients for a decade. In 2017, the results from KEYNOTE-045, an open-label, international, phase 3 clinical trial, demonstrated the survival benefit of pembrolizumab, the programmed cell death protein–1 (PD-1) antibody, compared with second-line chemotherapy (docetaxel, paclitaxel, and vinflunine) in patients with advanced platinum-refractory UC, which led to its approval by the FDA [2]. Since then, pembrolizumab has been widely used in a large number of patients worldwide. The updated results from the KEYNOTE-045 trial with >2 years follow-up exhibited one- and two-year overall survival (OS) rate of 44.2% and 26.9% in patients treated with pembrolizumab, with a relatively modest objective response rate (ORR) to this drug of 21.1% [3]. On the other hand, patients who achieved an objective response to pembrolizumab experienced a durable response with a median of >2 years, suggesting the distinct survival outcomes for patients treated with pembrolizumab. Therefore, the optimal risk stratification to predict survival outcomes at the initiation of pembrolizumab treatment would be helpful for physicians. In the present study, we examined a risk model developed using the predictive factors for OS in mUC patients treated with pembrolizumab with the multi-institutional dataset.

## 2. Materials and Methods

The present study was retrospectively designed using a multi-institutional dataset of Osaka Medical and Pharmaceutical University (Osaka, Japan) and the Jikei University School of Medicine (Tokyo, Japan) between January 2018 and December 2020. All the patients enrolled in the study were diagnosed with metastatic urothelial carcinoma (UC), including upper tract UC (UTUC) and bladder cancer (BC), following the disease progression using platinum-based chemotherapy. Inclusion criteria to the present study were as follows: patients who had one more measurable metastatic sites according to the response evaluation criteria in solid tumors (RECIST) version 1.1 (defined measurable lesion of ≥10 mm using spiral CT scan) [4] and had at least one radiographic examination during their follow-up; patients who had a blood examination at the initiation of pembrolizumab treatment; and patients who had no clinical record of comorbidities of immune disease, anticancer medications, and steroids at the initiation of pembrolizumab treatment. The present study was approved by the institutional review board at Osaka Medical College (IRB approval number: RIN–750–2571, date of approval: 24 January 2020), and the study was performed based on the principles of the World Medical Association Declaration of Helsinki [5]. Written informed consent from patients was obtained at the enrollment of the study after a full explanation of the purpose and nature of all procedures.

CT scans for detecting any findings suspected of disease progression were scheduled every six weeks during the follow-up, and treatment response of pembrolizumab was assessed by the response evaluation criteria in solid tumors version 1.1 and iRECIST [4,6]. Re-evaluation using MRI, bone scintigraphy, and positron emission tomography/computed tomography (PET/CT) was further performed when necessary for the definitive diagnosis of immune-confirmed disease progression (iCPD) [6]. In detail, iRECIST defines immuno-unconfirmed disease progression (iUPD) based on RECIST 1.1 principles. However, iUPD requires confirmation, which is done on the basis of observing either a further increase in size or the number of new lesions (iCPD). When progression is not confirmed, but instead tumor shrinkage occurs (compared with baseline), which meets the criteria of iCR, iPR, or iSD, then the bar is reset so that iUPD needs to occur again. The primary endpoint was overall survival. OS was calculated as the interval from the initiation of pembrolizumab treatment to the date of the last follow-up or deaths from any cause. The secondary endpoint was objective response rate (ORR) using the best overall response after pembrolizumab treatment, which was defined as the percentage of patients who achieved complete response (CR) or partial response (PR) according to the RECIST version 1.1 and iRECIST [4,6]. Pembrolizumab was administrated intravenously at a dose of 200 mg every three weeks as approved by the FDA [2]. Treatment-related adverse events (AE) were recorded according to the guidelines of the National Cancer Institute Common Terminology Criteria for Adverse Events (CTCAE) version 4.0. Discontinuation of pembrolizumab treatment due to disease progression or treatment-related adverse events was decided by the physician. Clinical variables in the present study involved age at the initiation of pembrolizumab treatment, sex, smoking status, the primary site of cancer at diagnosis, pathological examination at diagnosis, radical treatment prior to metastasis, best response during chemotherapy prior to pembrolizumab treatment, number of metastatic sites at the initiation of pembrolizumab treatment, Eastern Cooperative Oncology Group performance status (ECOG-PS) at the initiation of pembrolizumab treatment, neutrophil–lymphocyte ratio (NLR) at the initiation of pembrolizumab treatment, and the occurrence of treatment-related AE during follow-up.

For statistical analyses, a Chi-square test was performed to evaluate the distribution of each variable by a contingency table. Kolmogorov–Smirnov normality was performed to check normal distribution in continuous variables. The Student’s t-test was conducted to assess the difference between the variables. For variables with non-normal distribution, a Wilcoxon or Kruskal–Wallis test was conducted to examine the difference between the groups. Kaplan–Meier curves were calculated to estimate the survival ratio. The ability for outcome prediction of continuous variables in NLR was determined via receiver operating characteristic (ROC) curve analysis, and the optimal cut-off values were defined by the Youden index as the point maximizing the difference between true positive rate and false positive rate across all possible cut-point values [7,8]. A log-rank test was performed to define the clinical difference between categorized groups. For multivariate analysis to examine the association of variables with OS, Cox proportional hazard regression models were utilized to define covariate-adjusted hazard ratios (HR). All the statistical tests were two-sided with *p* < 0.05 considered to delineate statistical significance, which was performed using the JMP^®^ 13 (SAS Institute Inc., Cary, NC, USA) and GraphPad Prism software version 9.1.2 (GraphPad Software, La Jolla, CA, USA).

## 3. Results

The clinical variables of all patients are shown in Table 1. The median age at the initiation of pembrolizumab treatment was 72 years old. Median OS from the initiation of pembrolizumab treatment was 11.7 months. Median follow-up times were 8 and 4 months for the patients who were alive (*n* = 117, 55.2%) and deceased (*n* = 95, 44.8%) during the follow-up, respectively. All patients had one or more metastatic sites at the initiation of pembrolizumab treatment (single site: 108 patients, 50.9%; multiple sites: 104 patients, 49.1%). For patients with one metastatic site (108 patients, 50.9%), the location of the metastatic site was as follows: liver (3 patients, 1.4%), lung (24 patients, 11.3%), lymph node (57 patients, 26.9%), and other sites (24 patients, 11.3%). For patients with multiple metastatic sites (104 patients, 49.1%), the location of the metastatic sites was as follows: liver (36 patients, 17.0%), lung (45 patients, 21.2%), lymph node (84 patients, 39.6%), and other sites (27 patients, 12.7%). The best overall response after pembrolizumab treatment in the total cohort was CR in 9 (4.2%), PR in 47 (22.2%), SD in 48 (22.6%), and PD in 108 (51.0%) patients. Thus, the ORR and disease control rate in the total cohort was 26.4% (56 patients) and 49.0% (104 patients), respectively. Treatment discontinuation of pembrolizumab was recorded in 160 (75.5%) patients during the follow–up due to disease progression (135 patients: 63.7%) and intolerable AE (25 patients: 11.3%). Treatment-related AE (CTCAE grade 1–4) were observed in 78 (36.8%) patients, of which there were 27 (12.8%), 29 (13.8%), 19 (9.0%), and 3 (1.4%) patients with a CTCAE grade of 1, 2, 3, and 4, respectively.

**Figure 1:**
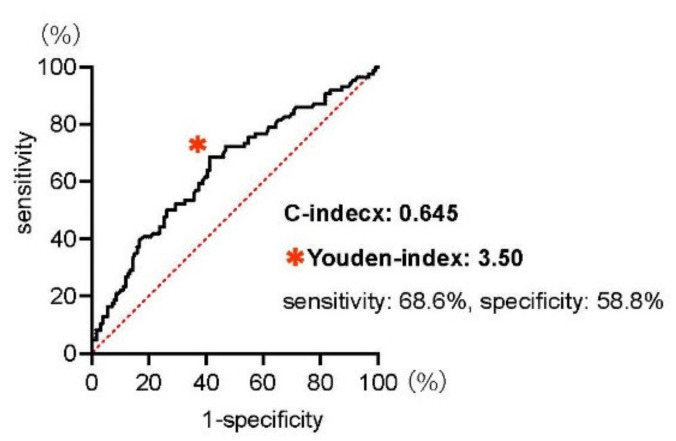
Receiver operating characteristic (ROC) curves for the neutrophil–lymphocyte ratio (NLR) at the initiation of pembrolizumab treatment for predicting one-year overall survival. The C-index was 0.645 (95%CI: 0.569–0.721), and the Youden index, the optimal cut–off value that maximizes the differences in sensitivity and specificity, was indicated at the * mark.

Since recent studies indicate the clinical utility of NLR as a predictive marker for patients treated with pembrolizumab [9], we examined the receiver operating characteristic (ROC) curve predicting the one-year OS in our multi-institutional dataset (Figure 1). The optimal cut-off value of NLR to predict the one-year mortality for patients treated with pembrolizumab was 3.50 as defined by the Youden index that maximizes the difference between true positive and false positive rates across all possible cut-point values (sensitivity: 68.6%, specificity: 58.8%), and the C-index was 0.645 (95%CI: 0.569–0.721).

**Figure 2:**
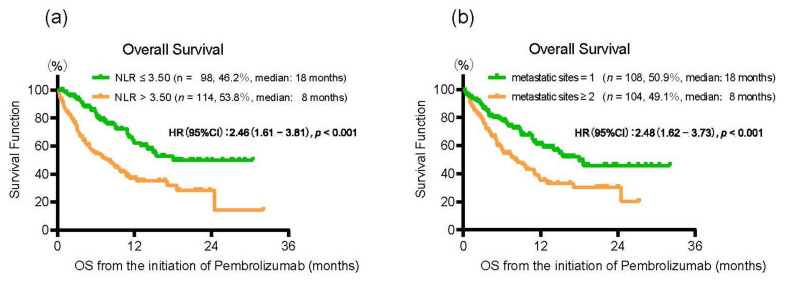
(**a**) Kaplan–Meier curves for overall survival (OS) from the initiation of pembrolizumab treatment according to the neutrophil–lymphocyte ratio (≤3.50/>3.50). (**b**) Kaplan–Meier curves for overall survival (OS) from the initiation of pembrolizumab treatment according to the number of metastatic sites (1/≥2).

Kaplan–Meier curves showed that patients with an NLR > 3.50 at the initiation of pembrolizumab treatment had a significantly shorter OS than those with NLR ≤ 3.50 (median OS of 8 and 18 months, HR: 2.46, 95% CI: 1.61–3.81, *p* < 0.001) (Figure 2a). To investigate the independent predictive factors for patients treated with pembrolizumab, we conducted a multivariate analysis for OS by using Cox proportional hazard regression models (Table 2). There were two clinical variables significantly associated with OS, including multiple metastatic sites (HR, 95% CI: 1.71, 1.07–2.77, *p* = 0.023) and NLR > 3.50 (HR, 95% CI: 2.20, 1.34–3.69, *p* = 0.001) at the initiation of pembrolizumab treatment. We confirmed that patients with multiple metastatic sites at the initiation of pembrolizumab treatment had a significantly shorter OS than those with a single metastatic site (median OS of 8 and 18 months, HR: 2.48, 95% CI: 1.62–3.73, *p* < 0.001) (Figure 2b).

**Figure 3:**
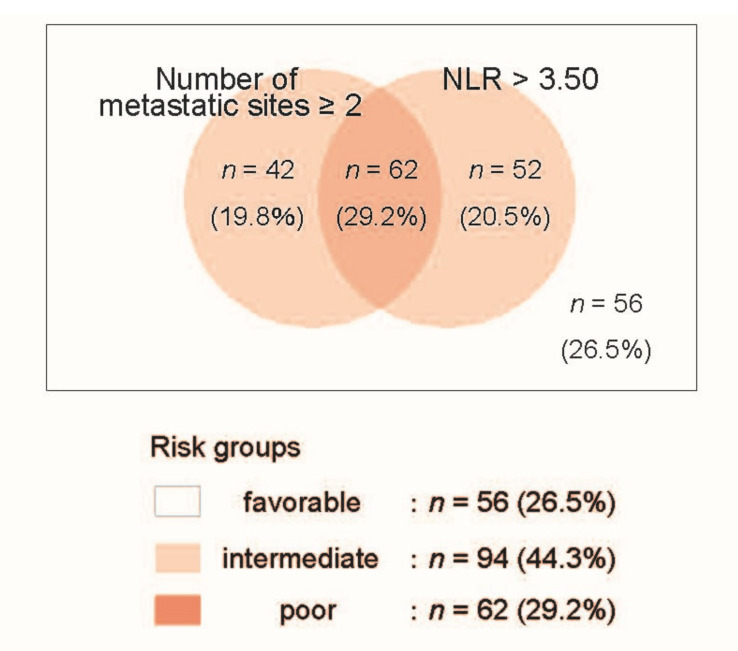
Venn diagram of the risk model using two risk factors, including the number of metastatic sites (1/≥2) and the neutrophil–lymphocyte ratio (≤3.50/>3.50) at the initiation of pembrolizumab treatment in 212 mUC patients.

Based on these results, we next developed a risk model to predict OS for patients treated with pembrolizumab using these two independent predictors, i.e., an NLR > 3.50 and multiple metastatic sites at the initiation of pembrolizumab treatment. Patients without any factors were assigned to the favorable-risk group (56 patients, 26.5%). Patients with either factor and with both factors were assigned to the intermediate-risk group (94 patients, 44.3%) and poor-risk group (62 patients, 29.2%), respectively (Figure 3).

**Figure 4:**
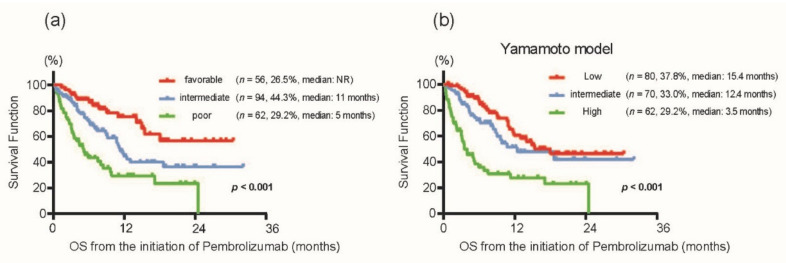
(**a**) Kaplan–Meier curves for overall survival from the initiation of pembrolizumab treatment according to the risk model in the present study. (**b**) Kaplan–Meier curves for overall survival from the initiation of pembrolizumab treatment according to the risk model from Yamamoto et al. [10] using our cohort.

Kaplan–Meier curves showed clear discrimination of OS among the risk groups (the median of “not reached” in the favorable-risk group, 11 months in the intermediate-risk group, and five months in the poor-risk group; *p* < 0.001) (Figure 4a). The ORR in each risk group was 35.7% in the favorable-risk group, 27.7% in the intermediate-risk group, and 17.7% in the poor-risk group. We also utilized another risk model for advanced UC patients treated with pembrolizumab previously reported by Yamamoto et al. using four factors (ECOG-PS ≥ 2, without only lymph node metastasis, CRP > 0.56 mg/dL, and an NLR > 3.0 at the initiation of pembrolizumab treatment) [10]. As expected, with the classification using the model by Yamamoto et al. [10] in our cohort, Kaplan–Meier curves also showed a distinct OS of 15.4 months in the favorable-risk group, 12.4 months in the intermediate-risk group, and 3.5 months in the poor-risk group (*p* < 0.001) (Figure 4b). Importantly, the concordance index for predicting OS in the present study (0.659, 95% CI: 0.587–0.723) was comparable with that from Yamamoto et al. (0.643, 95% CI: 0.568–0.712) (*p* = 0.622), indicating the potential utility of the risk groups in the present study.

## 4. Discussion

As of now, pembrolizumab treatment serves as a new mainstay in patients with platinum-refractory mUC, even though the effect of this drug substantially differs among individuals. For instance, results from the KEYNOTE-045 trial with >2 years follow-up showed a modest progression-free survival of 2.1 months (95% CI: 2.0–2.2 months), ORR of 21.1% (95% CI: 16.4–26.5%), and disease control rate of 38.5% (95% CI: 32.7–44.6%) with pembrolizumab treatment [3]. However, patients who achieved clinical response (CR and PR) to pembrolizumab appeared to exhibit a durable response (median duration of response of >24 months in these patients), which resulted in the longer median OS of 10.1 months (95% CI: 8.0–12.3 months) over the second-line chemotherapy (median OS of 7.3 months). Given the heterogeneous effect in the clinical outcomes, it is plausible to define the response marker to predict the treatment outcomes of using pembrolizumab.

To date, several clinicopathological factors have been proposed as prognostic indicators. Recent studies indicate that the NLR could serve as a therapeutic biomarker for mUC patients treated with pembrolizumab [11]. Interestingly, the NLR has also been reported as a potential marker for clinical survival in patients treated with immune checkpoint inhibitors (ICIs) for other malignancies, including renal cell carcinoma (RCC), non-small-cell lung cancer (NSCLC), and melanoma [12,13,14,15]. Nevertheless, the optimal risk stratification to predict survival outcomes at the initiation of pembrolizumab treatment is still controversial. Recently, Kobayashi et al. [9] reported a result from a large-scale database that consisted of 463 (discovery cohort) and 292 (validation cohort) mUC patients treated with pembrolizumab [9]. In their study, the median OS rates were 10.2 and 12.5 months in the discovery and validation cohorts, respectively (11.7 months in the present study). They developed a risk model using four factors, including performance status, hemoglobin level, site of metastasis, and the NLR at the initiation of pembrolizumab treatment. The patient distribution and median OS in each risk group in their discovery cohort were 119 patients (25.7%, the median OS: NR), 321 patients (69.3%, the median OS: 6.8 months), and 23 patients (5.0%, the median OS: 2.3 months) in the favorable-risk, intermediate-risk, and poor-risk groups, respectively. In the present study, we examined a risk model using the independent predictors for shorter OS, including an NLR > 3.50 and the presence of multiple metastatic sites at the initiation of pembrolizumab treatment. As shown in the Benn diagram in Figure 3, patients without any factors were assigned to the favorable-risk group. Patients with either factor and both factors were assigned to the intermediate-risk group and poor-risk group, respectively. This risk stratification seemed to be well-balanced (26.5%, 44.3%, and 29.2% in the favorable-risk group, intermediate-risk group, and poor-risk group, respectively), and Kaplan–Meier curves illustrated clear discrimination of OS among the risk groups (the median of “not reached” in the favorable-risk group, 11 months in the intermediate-risk group, and five months in the poor-risk group; *p* < 0.001). In addition, the ORR in each risk group was differentially distributed in the favorable-risk (35.7%), intermediate-risk (27.7%), and poor-risk (17.7%) groups.

The results from this research should be interpreted considering several limitations. First, we did not investigate the PD-L1-related investigation. Technically, the PD-L1 protein expression is determined using the 22C3 pharmDx (Agilent Technologies, Santa Clara, California, USA) by Combined Positive Score (CPS), which is the number of PD-L1 staining cells (tumor cells, lymphocytes, macrophages) divided by the total number of viable tumor cells, multiplied by 100. In 2020, the result of KEYNOTE-052 investigating the first-line pembrolizumab treatment in cisplatin-ineligible patients with locally advanced or metastatic urothelial carcinoma showed a significantly higher ORR of 47.3% in 110 patients with CPS ≥ 10 than ORR of 20.3% in 251 patients with CPS < 10 [16]. However, in Japan, pembrolizumab is currently approved only in the second-line setting after diagnosis of mUC. Due to the inconsistent protocol of immunohistochemistry among the institutes, we could not assess the clinicopathological value of PD-L1. Second, the present study was conducted using a retrospective design, and follow–up duration was relatively short compared with KEYNOTE–045 clinical trials. Third, radiographic and pathological diagnoses were not centralized. Lastly, discontinuation of pembrolizumab treatment was not standardized among the institutes throughout the study. Large-scale and prospective studies are further warranted to prove the results of the current study.

## 5. Conclusions

Our risk group stratification could precisely predict patient survival at the initiation of pembrolizumab treatment in mUC patients. Given that the model can be concisely determined at the initiation of pembrolizumab treatment, physicians may be encouraged to consider the risk group for daily practice.

## Figures and Tables

**Table 1:** Clinical variables in 212 patients with advanced platinum-resistant mUC.

Clinical Variables	Total (*n* = 212)	
	*n*	%
Age at the initiation of pembrolizumab treatment (years)		
Median (IQR)	72 (66–78)	
≤70	87	41.0
>70	125	59.0
Sex		
Male	151	71.2
Female	61	28.8
Smoking status at the initiation of pembrolizumab treatment		
Not current	186	87.7
Current	26	12.3
Primary site of cancer at diagnosis		
BT	130	61.3
UTUC	82	38.7
Pathological examination at diagnosis		
Pure UC	206	97.2
Not pure UC	6	2.8
Radical treatment prior to metastasis		
−	105	49.5
+	107	50.5
Best response during chemotherapy prior to pembrolizumab treatment (RECIST)		
Unknown	34	
ORR (−)	112	62.9
ORR (+)	66	37.1
Prior chemotherapy before pembrolizumab treatment		
GC	150	70.8
GCarbo	35	16.5
GCP	12	5.6
Others	15	7.1
Number of metastatic sites at the initiation of pembrolizumab treatment		
1	108	50.9
≥2	104	49.1
Location of metastatic site		
Liver	39	18.4
Lung	69	32.5
Lymph node	141	66.5
Others	42	19.8
ECOG-PS at the initiation of pembrolizumab treatment (0–4)		
0	92	43.4
≥1	120	56.6
NLR at the initiation of pembrolizumab treatment		
Median (IQR)	3.64 (2.36–5.76)	
Treatment–related AEs during follow–up (grade 0–4)		
0–2	189	89.2
≥3	23	10.8
OS from pembrolizumab treatment (months)		
Median	11.7	
6, 12, 18 months OS rate (%)	67.5, 48.4, 39.8	

mUC: metastatic urothelial carcinoma, BT: bladder cancer, UTUC: upper tract urothelial carcinoma, UC: urothelial carcinoma, ORR: objective response rate, RECIST: response evaluation criteria in solid tumors, GC: gemcitabine and cisplatin, GCarbo: gemcitabine and carboplatin, GCP: gemcitabine and cisplatin and paclitaxel, ECOG-PS: Eastern Cooperative Oncology Group performance status, NLR: neutrophil–lymphocyte ratio, AEs: adverse events, OS: overall survival, IQR: interquartile range.

**Table 2:** Multivariate analysis for OS from the initiation of pembrolizumab treatment in 212 mUC patients.

Clinical Variables	Multivariate Analysis		
	HR	(95% CI)	*p*-Value
Age at the initiation of pembrolizumab treatment (years)			
≤70/>70	1.08	(0.66–1.80)	0.734
Sex			
Male/Female	0.83	(0.47–1.43)	0.529
Smoking status at the initiation of pembrolizumab treatment			
Not current/Current	1.03	(0.47–2.08)	0.931
Primary site of cancer at diagnosis			
BT/UTUC	1.05	(0.63–1.70)	0.839
Pathological examination at diagnosis			
Pure UC/Not pure UC	1.98	(0.30–7.21)	0.408
Radical treatment prior to metastasis			
−/+	1.00	(0.62–1.61)	0.980
Best response during chemotherapy prior to pembrolizumab treatment (RECIST)			
ORR (−)/ORR (+)	1.25	(0.76–2.05)	0.362
Number of metastatic sites at the initiation of pembrolizumab treatment			
1/≥2	1.71	(1.07–2.77)	0.023 *
ECOG-PS at the initiation of pembrolizumab treatment (0–4)			
0/≥1	1.17	(0.71–1.96)	0.531
NLR at the initiation of pembrolizumab treatment			
≤3.50/>3.50	2.20	(1.34–3.69)	0.001 *
Treatment–related AEs during follow–up grade (0–4)			
0–2/≥3	0.70	(0.25–1.63)	0.442

OS: overall survival, BT: bladder cancer, UTUC: upper tract urothelial carcinoma, mUC: metastatic urothelial carcinoma, UC: urothelial carcinoma ORR: objective response rate, RECIST: response evaluation criteria in solid tumors, ECOG-PS: Eastern Cooperative Oncology Group performance status, NLR: neutrophil–lymphocyte ratio, AEs: adverse events, HR: hazard ratio, CI: confidence interval, * denotes *p* < 0.05.

## Data Availability

No new data were created or analyzed in this study. Data sharing is not applicable to this article.
